# Tumor PD-L1 expression and molecular profiling are not associated with immune checkpoint inhibitor-induced thyroid dysfunction in advanced NSCLC patients

**DOI:** 10.3389/pore.2023.1610951

**Published:** 2023-04-17

**Authors:** Adi Horesh, Rena Pollack, Hovav Nechushtan, Rivka Dresner-Pollak, Tzahi Neuman

**Affiliations:** ^1^ The Faculty of Medicine, Hebrew University of Jerusalem, Jerusalem, Israel; ^2^ Department of Endocrinology and Metabolism, Hadassah-Hebrew University Medical Center, Jerusalem, Israel; ^3^ Department of Oncology, Hadassah-Hebrew University Medical Center, Jerusalem, Israel; ^4^ Department of Pathology, Hadassah-Hebrew University Medical Center, Jerusalem, Israel

**Keywords:** PD-L1, NSCLC, immune checkpoint inhibitors, immune-related adverse events, thyroid

## Abstract

**Background:** Immune-checkpoint inhibitors (ICIs) have revolutionized the treatment of advanced non-small cell lung cancer (NSCLC), however are frequently associated with thyroid immune-related adverse events (IRAEs). We investigated the association between patient characteristics, tumor PD-L1 expression and molecular profile with the development of thyroid IRAEs in NSCLC patients.

**Methods:** Single center, retrospective study including 107 NSCLC patients treated with PD-1/PD-L1 inhibitors from April 2016 to July 2020. All patients were euthyroid at baseline with at least two TSH measurements post-treatment initiation. The primary outcome was the difference in tumor PD-L1 expression in patients who developed any thyroid IRAEs versus those who remained euthyroid. Additional outcomes included development of overt thyroid dysfunction, the association of specific molecular alterations with thyroid IRAEs, and onset of thyroid IRAEs as a function of tumor PD-L1 expression.

**Results:** Overall, 37 (34.6%) patients developed any thyroid dysfunction and 18 (16.8%) developed overt thyroid dysfunction. Tumor PD-L1 staining intensity was not associated with thyroid IRAEs. TP53 mutation was less likely to be associated with any thyroid dysfunction (*p* < 0.05) and no association was found between EGFR, ROS, ALK or KRAS mutations. There was no association between PD-L1 expression and time to develop thyroid IRAEs.

**Conclusion:** PD-L1 expression is not associated with the development of thyroid dysfunction in advanced NSCLC patients treated with ICIs, suggesting that thyroid IRAEs are unrelated to tumor PD-L1 expression.

## Introduction

Immune checkpoint inhibitors (ICIs) have revolutionized the management of advanced non-small cell lung cancer (NSCLC). Specifically, programmed cell death 1 (PD-1) and programmed cell death ligand 1 (PD-L1) inhibitors as monotherapy or in combination with chemotherapy are associated with improved response rates and overall survival compared with conventional chemotherapy (1). Thus, ICIs are now widely used as first line monotherapy in patients with advanced stage NSCLC and >50% membranous PD-L1 positive tumor cells and in combination with chemotherapy irrespective of PD-L1 expression (2). In the setting of specific oncogenic driver mutations such as anaplastic lymphoma kinase (ALK), v-Raf murine sarcoma viral oncogene homolog B1 (BRAF), epithelial growth factor receptor (EGFR), c-ros oncogene 1 (ROS1) and neurotrophic tyrosine receptor kinase (NTRK) targeted therapies are recommended.

While the use of ICIs has provided significant benefit with an improved side effect profile, their use has been associated with the development of immune-related adverse events (irAEs). These irAEs have been known to affect all organ systems, however frequently result in thyroid dysfunction with a reported incidence of 9%–42% in patients treated with PD-1 or PD-L1 inhibitor therapy (3–7). Importantly, the development of irAEs has been associated with improved clinical outcomes (7–10).

The mechanism of thyroid irAEs has yet been understood and the underlying causes for the thyroid gland to frequently become affected remain elusive. Several studies have suggested that the thyroid dysfunction seen with ICI use is a manifestation of destructive thyroiditis due to unleashing cytotoxic T cells against thyroid antigens. This leads to humoral immune response which produces anti-thyroid antibodies (11). Another hypothesis is that ICI-induced thyroid dysfunction may be due to unmasking of latent autoimmunity in those with a predisposition to develop autoimmune disease (12–14). Interestingly, a recently published study suggests that patients without thyroid autoantibodies are also at increased risk of developing thyroid irAE after treatment with a combination of PD-1/CTLA-4-Ab compared to PD-1 Ab-alone, and this risk is similar to the risk of thyroid irAE in patients with ATA at baseline after PD-1 Ab therapy (15).

Several predictors of thyroid irAE development have been proposed including the presence of thyroid autoantibodies at baseline, higher TSH at baseline, increased BMI, and duration of treatment (13, 16–19). Data regarding the relationship between tumor characteristics and risk of irAEs are limited. In the present study, we aimed to explore the association between patient factors, tumor PD-L1 expression and molecular profile and the development of thyroid irAEs in NSCLC patients treated with PD-1 or PD-L1 inhibitors.

## Materials and methods

We conducted a non-interventional retrospective study utilizing deidentified data from the Hadassah Hebrew-University Medical Center clinical and pathologic database. Complete demographic, clinical and pathologic data are recorded in the Hadassah Hebrew-University Medical Center electronic medical records (EMR), including medical diagnoses, laboratory tests, pathology data, prescribed medications, and information on all patient medical interactions including outpatient visits, acute medical services, and hospitalizations. Approval was obtained from the Hadassah Medical Center Institutional Review Board and Ethics Committee for the purpose of accessing and analyzing the data. Individual patient informed consent was not required because of the anonymized nature of the patient records and the non-interventional study.

### Study population

The present study included patients aged 18 years or above with biopsy proven advanced NSCLC and PD-L1 tumor expression data from 1 April 2016 to 30 July 2020. Patients meeting inclusion criteria were ≥18 years old with biopsy-proven NSCLC and PD-L1 tumor immunohistochemistry (IHC) staining results, who initiated treatment with single agent PD-1 inhibitor (nivolumab, pembrolizumab) or PD-L1 inhibitor (durvalumab, atezolizumab) according to standard protocols. Thyroid function tests (TFTs) within 3 months prior to treatment initiation and at least 2 measurements thereafter were required. Subjects were excluded if they had known history of thyroid dysfunction, including prior diagnosis of thyroid disease, past treatment with antithyroid drugs or levothyroxine, or evidence of abnormal thyroid function tests at baseline. Patients were censored at the date of last contact or date of death.

### Assessment of outcome

The relationship between PD-L1 tumor staining and each of the clinical outcomes was assessed. The primary outcome was defined as the association of tumor PD-L1 expression in patients developing any thyroid dysfunction versus remaining euthyroid. Additional outcomes included: 1) the association of PD-L1 expression with the development of overt thyroid dysfunction versus remaining euthyroid 2) the association of specific molecular alterations (EGFR, ALK, ROS, tumor protein 53 (TP53) and Kirsten rat sarcoma (KRAS) with the development of any thyroid dysfunction versus remaining euthyroid, and 3) time to development of thyroid dysfunction as a function of tumor PD-L1 expression.

### Variables, definitions, derivations, and measurements

Clinical data collected included age, sex, smoking status, type and stage of malignancy, previous chemotherapy or radiation, type of ICI agent, line of treatment, thyroid function tests at baseline and all available measurements post ICI treatment. Pathologic data including the biopsy method, pathologic diagnosis, tumor PD-L1 expression and molecular profiling was also collected.

Each tissue sample was evaluated morphologically and with immunostaining when required for diagnostic purposes. Following the common guidelines, only two immunostains were performed (TTF1 and P40) to preserve tissue for additional evaluation (20). If the specific diagnosis was not definitive, the case was concluded as “NSCLC.” Once the pathologic diagnosis was determined, each advanced-stage sample was followed routinely by IHC for PD-L1 and molecular profiling.

### Immunohistochemistry staining

PD-L1 IHC staining was performed with 22C2 PharmDx, Dako, concentrated antibody, 1:33 dilution), using the automated staining platform (Ventana BenchMark Ultra), according to the Israeli harmonization protocol (21). The tissue blocks were cut at 5-µm thick sections. On each slide, a section of benign tonsillar tissue was used as a positive external control to evaluate the proper dynamic range of staining. In addition, positive histocytes involved within the tissue in each section was used as a positive internal control. Pathologists experienced in PD-L1 interpretation evaluated all the stained material. The samples were divided into 3 categories of staining intensity: negative (<1% membranous PD-L1 positive cells), weakly positive (1%–49% membranous PD-L1 positive cells), and strongly positive (≥50% membranous PD-L1 positive tumor cells) following the established protocol in the Keynote 024 clinical trial (1).

### Molecular profile

Molecular profiling from 2016 to 2017 included EGFR alterations status (Cobas® EGFR mutation kit; Cobas® z 480 analyzer), ALK (D5F3 antibody; Ventana BenchMark XT) and ROS (D4D6 antibody; ULTRA) immunostains. In 2018, the Cobas® system was replaced by a 24-gene panel, including EGFR, BRAF, KRAS and TP53 (Ion Torrent System; Oncomine™ Solid Tumor DNA Kit). As of 2020, comprehensive genomic profiling is performed using a next-generation sequencing assay including more than 500 relevant genes for tumorigenesis (Ion Torrent system; Oncomine™ Comprehensive Assay Plus Kit; sequencing performed using Ion S5™ System).

Thyroid outcomes were defined as follows:

(1) Any thyroid dysfunction was defined as a TSH value above or below the laboratory-specific reference range, irrespective of FT4 and FT3 levels during follow-up.
(2) Overt thyroid dysfunction was defined as either overt hypothyroidism, overt thyrotoxicosis, or central hypothyroidism. Overt hypothyroidism was defined as either elevated TSH with decreased FT4 and/or FT3 or TSH ≥10 mU/L. Overt thyrotoxicosis was defined as a suppressed TSH with elevated FT4 and/or FT3 levels. Central hypothyroidism was defined as a TSH value below the laboratory-specific reference range with a low FT4 and/or FT3 or a normal TSH value with a low FT4.
(3) Thyroiditis was defined as subclinical or overt thyrotoxicosis followed by progression to overt hypothyroidism.

### Statistical analysis

The association between two categorical variables was tested by applying either the Chi-square test or the Fisher’s exact test. Comparing quantitative variables between two independent groups was carried out using the t-test or the non-parametric Mann-Whitney test. The non-parametric test was used for data which was not normally distributed. A *p*-value of 0.05 or less was considered statistically significant. Analyses were performed using the IBM SPSS statistics software, version 25.

## Results

### Study cohort

**FIGURE 1:**
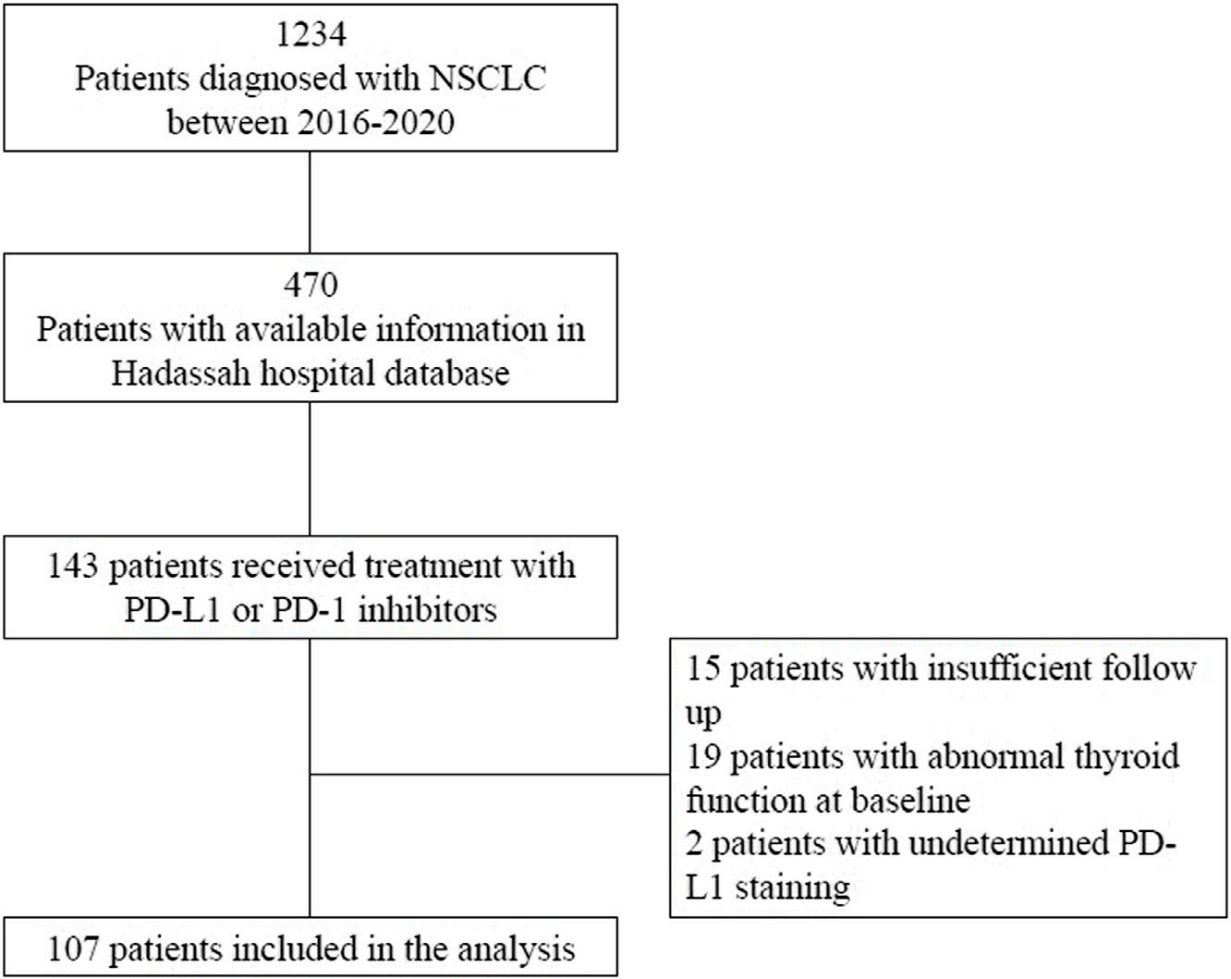
Consort Diagram. Abbreviations: NSCLC, Non-small cell lung cancer; PD-1, programmed cell death 1; PD-L1, programmed cell death ligand 1.

We identified 1,234 patients diagnosed with biopsy proven non-small cell lung cancer (NSCLC) from 1 April 2016 to 30 July 2020 in the Hadassah-Hebrew University pathology database. Of these, 470 patients had demographic, clinical and pathologic data available in the Hadassah-Hebrew University Medical Center EMR. A total of 143 patients received treatment with a PD-1 or PD-L1 inhibitor. After applying all inclusion and exclusion criteria, 107 patients were included in the study and followed for a median 371 (IQR 169.8–620.0) days (Figure 1).

Baseline characteristics of the overall cohort are presented in Table 1.

**TABLE 1:** Baseline characteristics of the overall cohort.

Variable	N = 107
Age, y ± SD	66.1 ± 9.6
Sex	
Male	78 (72.9)
Female	29 (27.1)
Smoking	88 (82.8)
BMI, kg/m^2^ ± SD	24.5 ± 3.7
Lung Malignancy	
SCC	27 (25.2)
Adenocarcinoma	63 (58.9)
NSCLC	17 (15.9)
Tumor Stage	
II/III	23 (21.5)
IV	86 (78.5)
ICI type	
PD-1	98 (91.6)
PD-L1	9 (8.4)
Line of treatment	
1	49 (45.8)
2	49 (45.8)
≥3	9 (8.4)
Prior chemotherapy	79 (73.8)
Prior of radiation	64 (59.8)
Baseline TSH, mU/L± SD	1.6 ± 0.9

Continuous parameters are shown as mean ± SD. Categorical variables are shown as n (%) of the overall population. Abbreviations: BMI, Body Mass Index; SCC, Squamous Cell Carcinoma; NSCLC, Non-small cell lung cancer; ICI, Immune-checkpoint inhibitor; PD-1, programmed cell death 1; PD-L1, programmed cell death ligand 1; TSH, Thyroid-Stimulating Hormone.

The study population consisted of 72.9% men, 82.2% smokers, with mean age 66.1 ± 9.6 years. Overall, 78.5% were diagnosed at stage IV and 58.9% had adenocarcinoma of the lung. Mean baseline TSH was 1.6 ± 0.9 mU/L.

Biopsies were performed using transthoracic needle biopsy in 46 (42.9%) patients, transbronchial biopsy in 24 (22.4%) patients, endobronchial ultrasound-guided biopsy in 14 (13.1%) patients, and 10 patients (9.4%) underwent open biopsy.

### Outcomes

#### Any thyroid dysfunction

A total of 37 (34.6%) patients developed any thyroid dysfunction. Patients developing any thyroid dysfunction were of similar age, gender, and smoking status compared to those who remained euthyroid. Furthermore, baseline TSH was similar between the two groups (Table 2).

**TABLE 2:** Demographic and clinical characteristics associated with the development of any thyroid dysfunction.

	Euthyroid (N = 70)	Any thyroid dysfunction (N = 37)	*p*-Value
Age, y± SD	65.5 ± 10.3	67.1 ± 8.2	0.41
Male (*n* = 78)	49 (62.8)	29 (37.2)	0.35
Lung Malignancy			0.59
SCC (*n* = 27)	19 (70.4)	8 (29.6)	
Adenocarcinoma (*n* = 63)	41 (65.1)	22 (34.9)	
NSCLC (*n* = 17)	10 (62.5)	7 (37.5)	
Tumor Stage			
II/III (*n* = 23)	15 (65.2)	8 (34.8)	0.98
IV (*n* = 86)	55 (65.5)	29 (34.5)	
ICI type			
PD-1 (*n* = 98)	66 (67.3)	32 (32.7)	0.27
PD-L1 (*n* = 9)	4 (44.4)	5 (55.6)	
Prior chemotherapy (*n* = 79)	49 (62.0)	30 (38.0)	0.22
Prior radiation (*n* = 64)	42 (65.6)	22 (34.4)	0.96
Baseline TSH, mU/L	1.6 ± 0.8	1.6 ± 1.1	0.82

Continuous parameters are shown as mean ± SD. Categorical variables are shown as n (%) of the overall population of each row. SCC, Squamous Cell Carcinoma; NSCLC, Non-small cell lung cancer; ICI, Immune-checkpoint inhibitor; PD-1, programmed cell death 1; PD-L1, programmed cell death ligand 1; TSH, Thyroid-Stimulating Hormone. *p*-value is shown for comparison of euthyroid patients (*n* = 70) versus all patients with any thyroid dysfunction (*n* = 37). Chi square test and Fisher’s exact test were applied in the comparison of categorical variables; T-test or Mann-Whitney test were used to compare quantitative variables.

Of those that developed any thyroid dysfunction, 13 (35.1%) had negative PD-L1 IHC staining, 9 (24.3%) were weakly positive and 15 (40.5%) were strongly positive. No difference was noted in PD-L1 IHC staining intensity between those that developed any thyroid dysfunction vs. remained euthyroid (Table 3).

**TABLE 3:** Pathologic characteristics associated with the development of any thyroid dysfunction.

	Overall	Euthyroid (N = 70)	Any thyroid dysfunction (N = 37)	*p*-value
PD-L1 (N = 107)				
Negative	33 (30.8)	20 (60.6)	13 (39.4)	0.22
Weakly positive	19 (17.8)	10 (52.6)	9 (47.4)	
Strongly positive	55 (51.4)	40 (72.7)	15 (27.3)	
EGFR mutated (N = 98)	10 (10.2)	9 (90.0)	1 (10.0)	1.00
ALK mutated (N = 52)	0 (0.0)	0 (0.0)	0 (0.0)	1.00
ROS mutated (N = 46)	1 (2.2)	1 (100.0)	0 (0.0)	0.66
TP53 mutated (N = 92)	46 (50.0)	36 (78.3)	10 (21.7)	0.01
KRAS mutated (N = 90)	27 (30.0)	18 (66.7)	9 (33.3)	1.00

Variables are shown as n (%) of the overall population in each row. *p*-value is shown for comparison of euthyroid patients (*n* = 70) versus all patients with any thyroid dysfunction (*n* = 37). Chi square test and Fisher’s exact test were applied in the comparison of categorical variables.

#### Overt thyroid dysfunction

Eighteen (16.8%) patients developed overt thyroid dysfunction, including 5 (4.7%) overt hypothyroidism, 7 (6.5%) overt thyrotoxicosis and 6 (5.6%) thyrotoxicosis with progression to overt hypothyroidism consistent with thyroiditis. No significant difference in baseline characteristics were noted between the two groups (Supplementary Table S1). Of those that developed overt thyroid dysfunction, no significant difference identified regarding the age, gender, specific diagnosis, tumoral stage, prior treatment (chemotherapy or radiation) or the specific ICI treatment. Regarding PD-L1 expression, 8 (44.4%) had negative PD-L1 IHC staining, 3 (16.7%) were weakly positive and 7 (38.9%) were strongly positive. PD-L1 IHC staining intensity was similar between patients that developed overt thyroid dysfunction vs. remained euthyroid (Supplementary Table S2).

#### Molecular markers

We further analyzed the relationship between any thyroid dysfunction and the presence of EGFR, ALK, ROS, TP53 and KRAS mutations. Overall, 10/98 (10.2%) patients tested positive for an EGFR mutation, 1/46 (2.2%) was positive for a ROS rearrangement, 46/92 (50.0%) had a TP53 mutation, and 27/90 (25.2%) patients were positive for a KRAS mutation. No patients were found to have an ALK rearrangement. There was no association found between the development of any thyroid dysfunction and EGFR, ROS, ALK or KRAS mutations, however patients with TP53 mutation were less likely to be associated with the development of any thyroid dysfunction (*p* < 0.05; Table 3). No association was noted between overt thyroid dysfunction and the presence of these mutations (Supplementary Table S2).

#### Time to development of any thyroid dysfunction

The median time to the development of any thyroid dysfunction among the entire cohort was 45.0 (IQR 29.5–91.0) days. There was no association between PD-L1 expression and time to develop thyroid dysfunction.

## Discussion

This single center, retrospective study assessed the association between clinical parameters, tumor PD-L1 expression, molecular profiling and the development of thyroid irAEs in NSCLC patients treated with PD-1 or PD-L1 inhibitors. Overall, 34.6% of patients developed thyroid dysfunction, including 16.8% of patients with overt thyroid dysfunction, consistent with previously reported studies (3–7). We did not find an association between baseline clinical parameters or tumor PD-L1 expression and the development of thyroid irAEs. TP53 mutation was associated with a decreased tendency to develop any thyroid dysfunction. The time to develop thyroid dysfunction was similar among the patients, regardless of PD-L1 expression.

Several studies have demonstrated a relationship between treatment response and the development of irAEs in NSCLC patients treated with immunotherapy (7, 9, 10). Specifically, thyroid dysfunction was associated with improved treatment response in NSCLC. In a subgroup analysis of patients with NSCLC treated with pembrolizumab in the KEYNOTE-001 trial, overall survival was significantly longer in subjects who developed ICI-induced thyroid dysfunction (14). Similarly, a study including patients with NSCLC, renal cell carcinoma and metastatic melanoma treated with anti PD-1 immunotherapy demonstrated improved progression-free survival (PFS) and overall survival (OS) in patients who developed thyroid dysfunction compared to patients without (22). It has therefore been proposed that irAEs, and specifically, thyroid dysfunction may serve as a surrogate marker for treatment response in patients treated with ICIs.

Considering the mechanism of ICI therapy, it seems reasonable that irAEs may be more likely to occur in patients with high tumor PD-L1 expression. The relationship between PD-L1 expression and irAEs has been examined, with discrepant results noted among studies. Sugisaka et. al performed a retrospective analysis of 44 patients with advanced NSCLC treated with PD-1 monotherapy (23). Overall, 70% of patients developed irAEs of any grade. Patients who developed irAEs demonstrated improved objective response rate (ORR), disease control rate and PFS. High PD-L1 expression (≥50%) was identified as an independent predictor of irAE development. In a recent real-world study including 877 patients with NSCLC and PD-L1 expression ≥50% treated with PD-1 monotherapy, endocrine irAEs were noted in 27% of patients (9). Patients who developed endocrine irAEs had improved ORR, PFS and OS in multivariate analyses. In contrast, a significant relationship between PD-L1 expression and irAE development was not found in a subgroup of patients treated with PD-1 inhibitor monotherapy in the KEYNOTE-001 trial (24). While 40% of patients developed irAEs associated with improved ORR, PFS, OS, no association with PD-L1 status or a targetable mutation (EGFR or ALK) was noted. Similarly, in the BIRCH trial which studied PD-L1 inhibitor treatment in NSCLC, a significant association between treatment response and PD-L1 expression was noted, however there was no association between PD-L1 expression and irAEs (25). In the current study, we did not demonstrate an association between PD-L1 expression and the thyroid irAEs suggesting that the development of thyroid irAEs is unrelated to PD-L1 expression in the tumor.

Even though no statistically significant correlation was shown for the classic molecular alterations, TP53 mutation was associated with a decreased tendency to develop any thyroid dysfunction following PD-1/L1 therapy. This phenomenon is interesting, with the understanding that not only does TP53 loss or mutation predispose to cancer, but hyperactive TP53 also drives various pathologies, including developmental phenotypes, premature aging, neurodegeneration, and side effects of cancer therapies (26). It has been previously shown that TP53 activation in response to genotoxic cancer therapies can promote side effects associated with DNA-damaging therapies (27). In our study, we demonstrate a decreased predisposition to endocrine side effects in patients treated with immunotherapy alone or with combined chemotherapy and a TP53 alteration. There was no association between PD-L1 expression and TP53 mutation in this cohort.

A biological rationale for this positive effect can be through the role of TP53 in regulating signaling within the tumor microenvironment, a function of great significance for antitumor immune responses, including both innate and adaptive immune responses (28). Thus, the subgroup of tumors with TP53 alteration might react differently with less of an immunomodulatory effect on the thyroid. However, additional data is required for further validation of this finding.

The strength of our study lies in our assessment of a large cohort of NSCLC patients with PD-L1 expression data, and for whom we have computerized access to all clinical, laboratory, and pathology data. Several limitations of our study are noted. First, it was retrospective, performed at a single institution which may introduce bias in the analysis. Furthermore, the number of patients with NSCLC treated with a PD-1 or PD-L1 inhibitor was limited and further examination in a larger population is warranted. In addition, not all patients had comprehensive molecular profiling, as it was not yet standard of care in the initial study period. Lastly, the relationship between PD-L1 tumor expression, thyroid irAEs and clinical response was not evaluated in this study.

In conclusion, our study does not demonstrate an association between high PD-L1 expression and thyroid irAEs in advanced NSCLC patients treated with immunotherapy. Further studies in a larger patient population are indicated to further understand the complex relationship between irAEs, tumor characteristics and treatment response.

## Data Availability

The original contributions presented in the study are included in the article/Supplementary Material, further inquiries can be directed to the corresponding author.

## Abbreviations

NSCLC, Non-small cell lung cancer; PD-1, programmed cell death 1; PD-L1, programmed cell death ligand 1.
