# Supplementary material for: Tumor PD-L1 expression and molecular profiling are not associated with immune checkpoint inhibitor-induced thyroid dysfunction in advanced NSCLC patients
Source: Pathol Oncol Res. 2023 Apr 17;29:1610951. doi: 10.3389/pore.2023.1610951 (PMC10149681; doi:10.3389/pore.2023.1610951)
Supplement: Supplementary file 1 [file Table1.docx]

**Table S1. Clinical characteristics associated with the development of overt thyroid dysfunction.**

|  | **Euthyroid**  **(N=70)** | **Overt Thyroid Dysfunction**  **(N=18)** | ***P* value** |
| --- | --- | --- | --- |
| Age, y± SD | 65.5 ± 10.3 | 65.0 ± 8.7 | 0.85 |
| Male | 49 (75.4) | 16 (24.6) | 0.14 |
| Malignancy |  |  |  |
| SCC | 19 (86.4) | 3 (13.6) |  |
| Adenocarcinoma | 41 (78.8) | 11 (21.2) | 0.54 |
| NSCLC | 10 (71.4) | 4 (28.6) |  |
| Tumor Stage |  |  |  |
| II/III | 15 (88.2) | 2 (11.8) | 0.51 |
| IV | 55 (77.5) | 16 (22.5) |  |
| ICI type |  |  |  |
| PD-1 | 66 (80.5) | 16 (19.5) | 0.60 |
| PD-L1 | 4 (66.7) | 2 (33.3) |  |
| Prior chemotherapy | 49 (77.8) | 14 (22.2) | 0.51 |
| Prior radiation | 42 (77.8) | 12 (22.2) | 0.60 |
| Baseline TSH, mU/L | 1.6 ± 0.8 | 1.5 ± 1.0 | 0.44 |

Continuous parameters are shown as mean ± SD. Categorical variables are shown as n (%) of the overall population. SCC, Squamous Cell Carcinoma; NSCLC, Non-small cell lung cancer; ICI, Immune-checkpoint inhibitor; PD-1, programmed cell death 1; PD-L1, programmed cell death ligand 1; TSH, Thyroid-Stimulating Hormone.

**Table S2. Pathologic characteristics associated with the development of overt thyroid dysfunction.**

|  | **Euthyroid**  **(N=70)** | **Overt thyroid dysfunction**  **(N=18)** | ***P* Value** |
| --- | --- | --- | --- |
| PD-L1 (N=88) |  |  |  |
| Negative | 20 (71.4) | 8 (28.6) | 0.36 |
| Weakly positive | 10 (76.9) | 3 (23.1) |  |
| Strongly positive | 40 (85.1) | 7 (14.9) |  |
| EGFR mutated (N=81) | 9 (90.0) | 1 (10.0) | 0.68 |
| ALK mutated (N=42) | 0 (0.0) | 0 (0.0) | 0.798 |
| ROS mutated (N=34) | 1 (2.2) | 0 (0.0) | 0.623 |
| TP53 mutated (N=76) | 36 (85.7) | 6 (14.3) | 0.16 |
| KRAS mutated (N=74) | 18 (81.8) | 4 (18.2) | 1.00 |

Variables are shown as n (%) of the overall population.
